# Spotlight on very-low-density lipoprotein as a driver of cardiometabolic disorders: Implications for disease progression and mechanistic insights

**DOI:** 10.3389/fcvm.2022.993633

**Published:** 2022-10-04

**Authors:** Hsiang-Chun Lee, Alexander Akhmedov, Chu-Huang Chen

**Affiliations:** ^1^Department of Internal Medicine, Division of Cardiology, Kaohsiung Medical University Hospital, Kaohsiung Medical University, Kaohsiung, Taiwan; ^2^Department of Internal Medicine, School of Medicine, College of Medicine, Kaohsiung Medical University, Kaohsiung, Taiwan; ^3^Lipid Science and Aging Research Center, College of Medicine, Kaohsiung Medical University, Kaohsiung, Taiwan; ^4^Institute/Center of Medical Science and Technology, National Sun Yat-sen University, Kaohsiung, Taiwan; ^5^Graduate Institute of Animal Vaccine Technology, National Pingtung University of Science and Technology, Pingtung, Taiwan; ^6^Center for Molecular Cardiology, University of Zurich, Schlieren, Switzerland; ^7^Vascular and Medicinal Research, Texas Heart Institute, Houston, TX, United States

**Keywords:** very-low-density lipoprotein, cardiovascular disease, triglycerides, metabolic syndrome, apolipoproteins, cardiometabolic disorders

## Abstract

Very-low-density lipoprotein (VLDL) is the only lipoprotein containing apolipoprotein B that is secreted from the liver, where VLDL is assembled from apolipoproteins, cholesterol, and triglycerides. The primary function of VLDL is to transport cholesterol and other lipids to organs and cells for utilization. Apart from its role in normal biologic processes, VLDL is also known to contribute to the development of atherosclerotic cardiovascular disease. Large VLDL particles, which are subclassified according to their size by nuclear magnetic resonance spectrometry, are significantly correlated not only with atherosclerosis, but also with insulin resistance and diabetes incidence. VLDL can also be subclassified according to surface electrical charge by using anion-exchange chromatography. The most electronegative VLDL subclass is highly cytotoxic to endothelial cells and may contribute to coronary heart disease. In addition, electronegative VLDL contributes to the development of atrial remodeling, especially in patients with metabolic syndrome, which is an established risk factor for atrial fibrillation. In this review, we focus on the VLDL subclasses that are associated with apolipoprotein alterations and are involved in cardiometabolic disease. The postprandial enhancement of VLDL’s pathogenicity is a critical medical issue, especially in patients with metabolic syndrome. Therefore, the significance of the postprandial modification of VLDL’s chemical and functional properties is extensively discussed.

## Introduction

### Composition of very-low-density lipoprotein

Very-low-density lipoprotein (VLDL) is a precursor to intermediate-density lipoprotein (IDL), which subsequently forms low-density lipoprotein (LDL). Density-gradient ultracentrifugation is the standard method used to isolate VLDL and other major lipoproteins, including chylomicrons, IDL, LDL, and high-density lipoprotein (HDL) from serum or plasma (1, 2). The lipid core of VLDL consists of triglycerides (TGs, 50–70% of particle mass), cholesterol ester (10–25%), and fatty acids (<10%). The major core protein of VLDL is apolipoprotein (apo)B100; other proteins include apoCI, apoCII, apoCIII, and apoE. These surface apolipoproteins also serve as ligands for cell-surface receptors and coordinators for lipolysis (3).

### The physiologic functions of very-low-density lipoprotein – More than a cargo carrier for lipids

VLDL functions as a cargo carrier, transporting cholesterol, TGs, and proteins to peripheral cells for essential bioactivities. In the liver, TGs and cholesterol are incorporated with apoB100, which affects the lipid abundance and size of secreted VLDL (4). After VLDL is secreted, it is hydrolyzed by lipoprotein lipase (LPL), which is present in the capillary endothelium or associated with VLDL receptors, and transformed into VLDL remnant and IDL. HDL then takes up apoCII from VLDL remnant and IDL, and cholesterol ester transfer protein (CETP) exchanges their TGs and phospholipids with cholesterol. IDL can be taken up by the liver via the LDL receptor or after being transformed into LDL upon losing apoE and TGs (3). VLDL is a TG-rich lipoprotein, and its assembly and metabolism are affected by insulin resistance and long-term nutrient excess (5). VLDL also modulates nitric oxide signaling, which is essential for vascular smooth muscle relaxation and blood pressure control (6). In addition, VLDL enhances phospholipase D activity by increasing cytosolic calcium levels and stimulates aldosterone synthesis in the adrenal gland (7). Therefore, VLDL does not only serve as a lipid cargo carrier, but it also modulates lipid-related blood pressure regulation.

### The classification of very-low-density lipoprotein by particle size

The diameter of VLDL particles can be measured using nuclear magnetic resonance (NMR) spectrometry. To classify VLDL subfractions by particle diameter, most studies have used a simplified classification system with different categories of average diameter. The quantitative analysis of serum or plasma lipoprotein subfractions requires high reproducibility. Such reproducibility has been examined by pooling quality control plasma lipoprotein samples and comparing NMR results among 11 spectrometers and 5 laboratories. In total, 16 subclasses were identified: 6 for VLDL, 6 for LDL, and 4 for HDL (8). However, a consensus has not been reached with respect to standard diameter ranges for classifying VLDL subfractions. For instance, in the study by Garvey et al. (9), three categories were defined as follows: large VLDL (>60nm), intermediate VLDL (35–60 nm), and small VLDL (<35 nm). In the study by Phillips et al. (10), the categories were defined as follows: large VLDL (including chylomicrons, if present, >60 nm), medium VLDL (42–60 nm), and small VLDL (<42 nm). Wang et al. (11) used six categories of VLDL as follows: largest (including chylomicrons, ± 75 nm), very large (average diameter, 64.0 nm), large (53.6 nm), medium (44.5 nm), small (36.8 nm), and very small (31.3 nm) VLDL.

### The classification of very-low-density lipoprotein by particle charge

In 1988, Avogaro et al. (12) first characterized LDL on the basis of surface electrical charge rather than particle size by using anion-exchange chromatography to separate LDL into LDL(+) and LDL(–). In addition, Yang et al. (13) and Chen et al. (14) divided LDL into five subfractions according to electrical charge, called L1-L5. Similarly, Chen et al. also used the same method of anion-exchange chromatography to separate VLDL into five subfractions, called V1-V5 (15) (Table 1).

**TABLE 1:** VLDL subclassified by size and electrical charge and the effects of VLDL subclasses on atherosclerotic CVD, MetS, and other conditions.

Classification	Patients	Fasting/postprandial	Effects	References
**NMR-based VLDL subclasses and atherosclerotic CVD**				
Large, medium, and small VLDL particles	Adults with incident coronary artery calcium (*n* = 6814; age, 45–85 years)	Overnight fasting (12 h)	Large VLDL was positively associated with incident coronary artery calcification in a model adjusted for scanner type, age, gender, and race	Zeb et al. (25)
Large, medium, and small VLDL particles	Healthy postmenopausal women (*n* = 286; mean age, 61.7 years)	Fasting (12 h)	Large VLDL was positively associated (*p* < 0.05) with higher coronary artery calcification after adjusting for age, systolic blood pressure, current smoking status, LDL cholesterol, HDL cholesterol, and triglycerides	Mackey et al. (26)
**NMR-based VLDL subclasses and MetS or other conditions**				
Large VLDL, medium VLDL, and small VLDL	Irish adults (*n* = 1834, middle-aged)	Overnight fasting	Metabolically healthy patients with smaller (below median) VLDL size	Phillips et al. (10)
Largest VLDL (including chylomicrons) and five different VLDL subclasses	Finnish men with or without glucose intolerance (*n* = 9399; mean age, 56.8 ± 6.9)	Overnight fasting	The concentrations of all lipid components in the VLDL subclasses were increased as glucose tolerance decreased	Wang et al. (11)
Large, intermediate, and small VLDL particles	Patients with or without diabetes (*n* = 148; mean age, 36.8 ± 11.8 years)	Overnight fasting	Progressive insulin resistance was associated with increased VLDL size and an increase in large VLDL particle concentrations	Garvey et al. (9)
Large, medium, and small VLDL particles	Healthy women (*n* = 26,836; age ≥ 45 years)	75.8% without-diabetes and 78.6% with diabetes were fasting	Large VLDL imparted a higher risk for incident type 2 diabetes mellitus than did small particles	Mora et al. (27)
	Women with type 1 diabetes mellitus (*n* = 112; mean age, 44.9 ± 7.8 years)	Overnight fasting (10–12 h)	Medium VLDL was associated with previous pre-eclampsia	Amor et al. (28)
Six VLDL subfractions (V1-V6, increasing density)	Adults, free of clinically detectable CVD (*n* = 6814; age, 44–84 years)	Fasting (12 h)	Several VLDL subfractions (V1-V4) were associated with abdominal body composition and intra-muscle fat infiltration	Marron et al. (91)
**Anion-exchange chromatography–based VLDL subclasses and MetS**				
VLDL subfractions with increasing negative charge (V1-V5)	Patients with or without MetS (*n* = 26)	Overnight fasting	V5, a highly negatively charged VLDL subfraction, directly damaged the endothelium	Chen et al. (15)
LDL and VLDL subfractions with increasing negative charge (L1-L5, V1–V5)	Asymptomatic individuals (*n* = 33; age, 32–64 years)	Fasting	Combined electronegativity of L5 and V5 plasma concentration was significantly correlated with coronary heart disease risk	Shen et al. (31)
Most electronegatively charged VLDL subfraction (VLDL-χ)	Patients with or without MetS (*n* = 167; age, 23–74 years)	Overnight fasting and postprandial	Plasma concentration of VLDL-χ (%) at 2 h postprandial was positively correlated with atrial enlargement in patients with MetS	Lee et al. (71)

CVD, cardiovascular disease; LDL, low-density lipoprotein; NMR, nuclear magnetic resonance; VLDL, very-low-density lipoprotein.

### Immunochemical isolation of very-low-density lipoprotein according to apolipoprotein content

Apolipoproteins are chemically unique, maintaining the structural integrity and functional specificity of different lipoprotein particles in lipid transport processes. Therefore, lipoproteins can be classified immunochemically according to their apolipoprotein composition (16). The two major classes of apolipoprotein-based families are apoA-containing and apoB-containing lipoproteins. VLDL, along with IDL and LDL, is an apoB-containing lipoprotein family. The apoB-containing lipoproteins can be divided into several subfamilies, including cholesterol ester-rich lipoprotein (LP-B) and TG-rich lipoproteins (16).

### Pathogenic very-low-density lipoprotein

The physiologic basis for the differences in composition, structure, and function among VLDL particles is important because these differences can strongly influence the atherogenic properties of VLDL. Moreover, abnormal VLDL can adversely affect vascular or cardiac cells (see below), which has important implications. In this review, we present a summary of the emerging evidence for VLDL in promoting cardiometabolic diseases and highlight how the subclassification of VLDL can be used to distinguish VLDL particles that are pathogenic from those that are physiologically necessary.

## Independent of low-density lipoprotein, very-low-density lipoprotein is associated with cardiometabolic disorders

### Cholesterols carried by both low-density lipoprotein and very-low-density lipoprotein are associated with atherosclerosis

Plasma LDL-cholesterol (LDL-C) alone is not sufficient to predict all non-atherosclerotic and atherosclerotic cardiovascular disease (ASCVD). Aside from LDL-C, VLDL cholesterol (VLDL-C) is also known to contribute to the development of ASCVD. Plasma VLDL-C is the primary component of non–HDL-cholesterol (HDL-C) (17) and is a predictor of ASCVD independent of LDL cholesterol (LDL-C) (18–20).

Prenner et al. (18) used cardiac electron beam computed tomography scanning to assess coronary artery calcification, which is an independent predictor of CVD risk, in a population of high-risk patients with type 2 diabetes. Their results showed that VLDL-C is an independent risk factor for coronary artery calcification, particularly in women. Furthermore, this association was independent of circulatory TG levels (18). In patients with type 2 diabetes who previously underwent coronary stent implantation, an elevated VLDL-C level >0.52 mmol/L was independently associated with in-stent restenosis (hazard ratio = 3.01) (21). Iannuzzi et al. (22) used ultrasound to measure carotid intima–media thickness in postmenopausal women and showed that VLDL-C was the lipoprotein most strongly associated with subclinical atherosclerosis. In addition, evidence from clinical studies has consistently indicated a causal role for TG-rich lipoproteins such as VLDL in ASCVD. An updated consensus statement regarding the current understanding of the role of TG-rich lipoproteins and their remnants in ASCVD has been published recently (5).

### The size of very-low-density lipoprotein affects its atherogenicity

Large VLDL particles have a greater association with the incidence of atherosclerosis than do smaller VLDL particles (Table 1). The size-based subclassification of lipoproteins is performed by the NMR analyzer, which uses characteristic signals of lipoprotein subclasses with different sizes as the basis for quantification. A set of purified standards is required for converting signal amplitudes to specific particle concentrations (23). The standards for VLDL are isolated by using a combination of ultracentrifugation and agarose gel filtration, and the size distribution is determined by using electron microscopy (2).

VLDL circulates in the blood for about 4 h before it is converted to IDL and then LDL (24). Lipolytic remodeling is responsible for the down-sizing of the largest VLDL particles and their conversion to IDL and LDL. Unlike small LDL, large VLDL was associated with an increased risk of incident coronary artery calcification and calcium score progression during follow-up (25). Likewise, in relatively healthy postmenopausal women, large VLDL was positively associated with coronary artery calcification, suggesting that the measurement of lipoprotein subclasses may improve the prediction of coronary artery disease beyond using the conventional lipid panel (26).

In addition, the size of VLDL was shown to be correlated with insulin resistance and diabetes mellitus (11, 27) (Table 1). In a prospective study by Mora et al. (27) of 26,836 initially healthy women followed for 13 years, large VLDL particles were found to predict type 2 diabetes. Likewise, Wang et al. (11) reported in a population study of 9399 Finnish men that abnormal glucose tolerance and new onset type 2 diabetes were associated with an increase in VLDL particles, with the exception of very small VLDL. Conversely, a lower number of large VLDL particles was shown to be the most significant predictor of metabolic health in adults, regardless of body mass index and obesity status (10). Garvey et al. (9) described the effects of insulin resistance and type 2 diabetes on the particle size and concentration of lipoprotein subclasses. Their results showed that progressive insulin resistance was associated with increased VLDL size. Compared with individuals who have normal insulin sensitivity, patients with insulin resistance or diabetes showed increased concentrations of large VLDL particles, but no change in medium VLDL or small VLDL particle concentrations. For patients with type 1 diabetes, medium VLDL particle concentration was independently associated with previous pre-eclampsia during pregnancy after adjusting for age and statin use (28).

### The charge-based electronegativity of very-low-density lipoprotein determines its atherogenicity

Lipoprotein particles can be separated according to charge by using anion-exchange chromatography. L5, which is the most electronegatively charged subfraction of LDL, induces endothelial apoptosis through the lectin-like oxidized LDL receptor-1 (LOX-1) in the absence of the LDL receptor (LDLR) (29). Similarly, the most electronegative subfraction of VLDL, V5, was shown to induce endothelial apoptosis and was the subfraction most rapidly internalized into endothelial cells (15). In addition, patients with metabolic syndrome (MetS) were found to have increased levels of electronegative VLDL. VLDL isolated from patients with MetS induced brain inflammation with glial cell activation in mice, suggesting that electronegative VLDL can promote cognitive dysfunction (30). Furthermore, Shen et al. (31) further confirmed that the most electronegative human plasma LDL (i.e., L5) and VLDL (i.e., V5) are highly atherogenic. In their study, the combined electronegativity of L5 and plasma concentration of V5 was significantly correlated with coronary heart disease risk in an age-adjusted analyses of asymptomatic individuals. Moreover, when human aortic endothelial cells were treated with L5 + V5 and L1 + V1, L5 + V5 induced significantly greater senescence-associated–β-galactosidase activity than did L1 + V1. In *ApoE^–/–^* mice, aortic lipid accumulation and cellular senescence were associated with the electronegativity of LDL and VLDL (31).

### Altered apolipoprotein content in very-low-density lipoprotein affects its atherogenicity

By 1972, the primary structures, including protein and DNA sequences, had been determined for almost all apolipoproteins (AI, AIV, B, CI, CII, CIII, D, E, I, and J) (16). VLDL particles containing apoE, apoCI, apoCIII, and apoAV have been shown to affect VLDL metabolism, site utilization, and atherogenicity. In the following sections, each lipoprotein is briefly described.

#### ApoE

Emerging evidence supports that the compositional change of apolipoproteins in VLDL affects its atherogenicity (Table 2). VLDL is one of several major lipoproteins containing apoE, which is a specific ligand for cysteine-binding repeats of the VLDL receptor (VLDLR). VLDLR is widely expressed throughout the body, including the heart, skeletal muscle, adipose tissue, and brain, and it has an important role in the uptake and metabolism of apoE-containing TG-rich lipoproteins. ApoE is a polymorphic protein arising from three alleles at a single gene locus (32). The enrichment of apoE content in VLDL has been shown to protect against coronary heart disease (33).

**TABLE 2:** Clinical studies showing altered VLDL apolipoproteins in patients with metabolic and atherogenic diseases.

Apolipoprotein	Study type	Patients	Fasting/ postprandial	Effects	References
ApoCI	Human	Cross-sectional studies (age, 56–80 years)	Fasting and postprandial (4 h)	ApoC1 positively correlated with carotid atherosclerosis	(35–37)
ApoCIII	Human	Ludwigshafen Risk and Cardiovascular Health Study (LURIC; *n* = 3041)	Not specified	Seven common variants of *APOC3* (rs734104, rs4520, rs5142, rs5141, rs5130, rs5128, and rs4225) were associated with modestly raised apoC-III and elevated VLDL/TG but were not associated with CAD	(92)
	Human	Middle-aged patients (*n* = 688; average age, 66 years; 52% women)	Fasting	ApoCII, apoCIII, and apoE were associated with composite CVD (fatal and non-fatal myocardial infarction, ischemic stroke, and sudden cardiac death)	(39)
ApoAV	Human	Patients with non-alcoholic fatty liver disease (*n* = 17) vs. healthy liver (*n* = 6)	Fasting	ApoA5 mRNA level was associated with hepatosteatosis	(46)
ApoE	Human	Two independent cohorts: women (*n* = 322; age, 30–55 years) and men (*n* = 418; age, 40–75 years)	Not specified	Increased apoE content in VLDL and LDL with apoCIII were associated with a lower risk of CHD	(33)
Angiopoietin-like protein (ANGPTL)-3	Human and mice	Humans and mice (e.g., *Angptl3*^–/^*^–^, Ldlr^–^*^/^*^–^, Lipg^–^*^/^*^–^*) with hyperlipidemia	Fasting	ANGPTL-3 inhibition reduces the content and size of lipids in VLDL	(54)

CAD, coronary artery disease; CHD, coronary heart disease; CVD, cardiovascular disease; LDL, low-density lipoprotein; TG, triglyceride; VLDL, very-low-density lipoprotein.

#### ApoCI

Primarily associated with HDL in the fasting state, apoCI transiently attaches to the surface of TG-rich lipoproteins such as chylomicrons and VLDL postprandially. ApoCI modulates several enzymes involved in lipoprotein metabolism and can reduce the uptake of VLDL by inhibiting its binding to VLDLR (34). The increased intima-media thickness of the common carotid artery indicates early atherosclerosis and was found to be associated with apoCI content in postprandial TG-rich lipoproteins (35, 36). In addition, the number of apoCI molecules per VLDL particle in the fasting state was associated with the plaque size of carotid atherosclerosis (37). ApoCI was also shown to be correlated with cholesterol enrichment in VLDL particles and the delayed clearance of TG-rich lipoproteins (37). In hypercholesterolemic rabbits, the constitutive expression of human apoCI provided protection against serious atherosclerosis (38). This benefit was found to be related to the inhibition of plasma cholesteryl ester transfer protein (CETP) activity (38). These findings support that apoCI enrichment attenuates the atherogenicity of VLDL particles.

#### ApoCIII

ApoCIII has been suggested to be a central regulator of TG-rich lipoprotein metabolism (39). A direct association of apoCIII with atherosclerosis was revealed by clinical genetic studies and studies showing that loss-of-function mutations in *APOC3* are associated with low TG levels (40) and a reduced incidence of ischemic CVD (41). Increased plasma levels of apoCIII are associated with increased levels of VLDL, IDL particles, and TGs (42). In human monocytic THP-1 cells, apoCIII activated protein kinase C alpha (PKCα) and transforming protein RhoA, which resulted in β1-integrin activation and promoted endothelial cell adhesion. These results suggested that apoCIII not only modulates lipoprotein metabolism, but may also contribute to atherosclerosis development (43). The antisense apoCIII inhibitor volanesorsen, which reduces apoCIII levels by >75% and plasma TGs levels, inhibits apoCIII synthesis in the liver (44). However, the indication for the clinical use of volanesorsen is limited to patients with familial chylomicronaemia syndrome for preventing pancreatitis; therefore, its effect on reducing CVD remains undetermined (39).

#### ApoAV

In contrast to *APOC3*, genotype combinations of common *APOA5* variants (c.-1131 T > C, S19 W, and c.*31C > T) are associated with elevated TG levels and increased CHD risk (45). In addition, patients with non-alcoholic fatty liver disease have elevated apoAV expression, which promotes hepatic TG storage in lipid droplets but decreases VLDL secretion by the liver (46). ApoAV also accelerates TG-rich lipoprotein uptake by the liver (47). However, the mechanism by which apoAV regulates circulatory VLDL metabolism remains largely unknown.

## Mechanisms of modified very-low-density lipoprotein in cardiometabolic disorders

### Overproduction of TGs in the liver and non-alcoholic fatty liver disease

**FIGURE 1:**
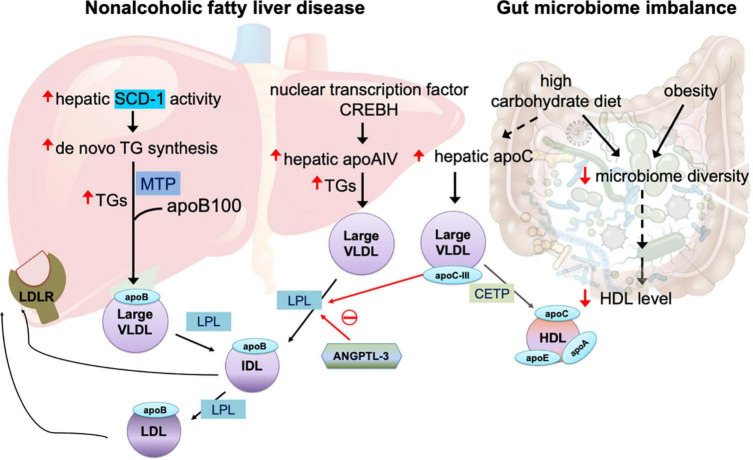
Mechanisms of large very-low-density lipoprotein (VLDL) in non-alcoholic fatty liver disease and gut microbiome imbalance. The overproduction of triglycerides (TGs) is related to increased activity of hepatic stearol-CoA desaturase (SCD)-1, which converts saturated fatty acids to monosaturated fatty acids that serve as the substrate for the synthesis of *de novo* TGs. The assembly of TGs with apolipoprotein (apo)B100 is facilitated by microsomal triglyceride transfer protein (MTP). In non-alcoholic fatty liver disease, the nuclear transcription factor cAMP-responsive element-binding protein H (CREBH) is upregulated, in turn increasing expression of hepatic apoAIV, which promotes the assembly of TG-rich, large VLDL. Angiopoietin-like protein family 3 (ANGPTL3) inhibits the enzyme activity of lipoprotein lipase (LPL), which is essential for breakdown of TGs in VLDL utilization. Both intermediate-density lipoprotein (IDL) and LDL particles are recognized by LDL receptor (LDLR) expressed in the liver. LPL activity is also inhibited by apoCIII. Large VLDL promotes plasma CETP-induced remodeling of TG-rich HDL. A high-carbohydrate diet and obesity impair microbiome diversity, which is related to reduced plasma HDL levels and increased hepatic apoCIII production that in turn inhibit LPL activity and enhance the abundance of large VLDL in the circulation.

A key feature of large VLDL is the overproduction of TGs in the liver, which may occur for several years before the onset of type 2 diabetes (27). In the liver, the biogenesis of VLDLs and the assembly of apolipoproteins are complex and highly regulated processes (4). A major source of TG synthesis is the endoplasmic reticulum (ER) lumen, where TGs are assembled with apoB100 to form lipid-poor primordial VLDL particles. This process is facilitated by microsomal triglyceride transfer protein (MTP) (4), which transfers both neutral and polar lipids to form VLDL particles (Figure 1). Whether and how MTP is modulated in patients with insulin resistance and diabetes remain unclear.

Because of its large size (average diameter >60 nm), VLDL is shifted from the ER membrane to the cis Golgi for cargo selection and vesicle formation. However, the utilization of vesicular carrier proteins for VLDL remains an ongoing subject of investigation (4). It has been suggested that VLDL exits the hepatic ER in a specialized vesicle (i.e., the VLDL transport vesicle), which can accommodate a particle diameter of up to 100–200 nm (48).

Patients with non-alcoholic fatty liver disease have increased hepatic stearol-CoA desaturase (SCD)-1 activity, which converts saturated fatty acids to monosaturated fatty acids that serve as a major substrate for the synthesis of *de novo* TGs and other lipids (49). How the abundance of TGs and the degree of TG desaturation are controlled or regulated during VLDL synthesis remain undetermined.

Hepatic apoAIV expression, which is regulated by nuclear transcription factor cAMP-responsive element-binding protein H (CREBH), is correlated with hepatic TG content in patients with chronic liver steatosis (50). CREBH activation plays key roles in hepatic steatosis by upregulating apoAIV during VLDL assembly in the ER and promotes the assembly of large and TG-enriched VLDL particles (50) (Figure 1). In addition to its expression in the liver, apoAIV is predominantly expressed in human enterocytes to facilitate intestinal chylomicron assembly and is highly upregulated after a fatty meal (51).

### Regulation of lipolysis

The utilization of VLDL and the breakdown of TGs in organs require the key enzyme lipoprotein lipase (LPL) to generate free fatty acids. The inhibition of lipolysis increases the size of circulating VLDL. Several members of the angiopoietin-like protein (ANGPTL) family regulate the activity of LPL. ANGPTL3, ANGPTL4, and ANGPTL8 are upregulated in patients with type 2 diabetes and obesity (52). In a group of patients who received RNA inhibition therapy with antisense oligonucleotides targeting *ANGPTL3*, protein levels of ANGPTL3 were reduced by as much as 84.5% from baseline 6 weeks after injection, while levels of TGs were reduced by 63.1%, VLDL cholesterol by 60.0%, and apoCIII by 58.8% (53). In mice, ANGPTL3 inhibition reduced TG content in the liver and retarded atherosclerosis progression (53). Endothelial lipase, which reduces LDL-C via an LDLR-independent mechanism, is essential for phospholipid reduction in VLDL and LDL (54). In *LDLR*^–/^*^–^* mice, ANGPTL3 inhibition caused a marked reduction in the TG content of VLDL. Furthermore, in *ApoE*^–/^*^–^* mice, ANGPTL3 inhibition promoted VLDL clearance with the involvement of multiple remnant receptors (54). However, in the liver, ANGPTL3 did not perturbate apoB lipidation and hepatic VLDL assembly (54). These findings suggest that ANGPTL3 governs VLDL catabolism and largely affects VLDL lipid content and size. On the other hand, endothelial lipase exerts anti-atherogenic effects by enhancing the catabolism of β-VLDLs (55), which are cholesterol-rich chylomicron and VLDL remnants that accumulate in the plasma of patients with type III dysbetalipoproteinemia (56). In elderly patients, the removal of TG-rich lipoprotein remnants is delayed, but TG breakdown is unchanged. Whether VLDL receptor function is impaired and whether ANGPTL3 is involved in aging-related, delayed VLDL removal remain unknown.

### Interaction of very-low-density lipoprotein with high-density lipoprotein

The reverse-remnant cholesterol transport mechanism, which is the acquisition of VLDL surface components by HDL during LPL-mediated lipolysis, plays an important role in VLDL catabolism (57). HDL affects the lipolysis of VLDL TGs and the release of surface lipids, free cholesterol, phospholipids, and exchangeable apoE, apoCII, and apoCIII from VLDL during lipolysis (58). HDL can also be classified into subpopulations according to size, apolipoprotein content, charge, mass, and density. Although subpopulations of both large and small HDL particles increased VLDL TG lipolysis efficiency and surface material removal from VLDL, the small, protein-enriched HDL particles exhibited a greater effect on this process and promoted a more efficient release of surface components, thereby affecting the properties of the generated remnants. Loss of apoC proteins from VLDL during lipolysis promoted the metabolism of apoB-containing lipoprotein because both apoCII and apoCIII inhibit the binding of apoB lipoproteins to the LDLR (58).

Increased TG content has been suggested to decrease the stability of HDL, VLDL, and LDL via several mechanisms. First, TGs have a direct destabilizing effect on lipoprotein particles from the CETP-induced remodeling of TG-rich HDL. Second, TGs have indirect effects that enhance spontaneous and enzymatic hydrolysis and oxidation. Third, products of the aforementioned processes, particularly free fatty acids, further augment lipoprotein destabilization and fusion. TGs are also involved in the substantial release of proteins from lipoproteins. Finally, the combination of destabilized LDL and VLDL enhances their retention in the arterial wall, triggering atherosclerosis (59).

### Genetic variants associated with very-low-density lipoprotein particles

Genetic variants have been associated with lipoprotein subclasses. Among those, the common variant rs73059724 resulted in small VLDL particles with fewer phospholipids (60). The variant rs73059724 is located on chromosome 19 and is associated with the promoter and intron of *HIF3A*, which regulates the cellular uptake of cholesterol esters and VLDL by promoting hypoxic conditions. In addition, *HIF3A* hypermethylation is associated with increased adiposity in Asian infants and children (61, 62). These findings suggest that HIF3A may regulate VLDL particle size. Furthermore, DNA methylation at *HIF3A* may explain the prenatal influences on adiposity. In another recent genetic study, Li-Gao et al. (63) investigated postprandial metabolomics and found that the *ANKRD55* locus led by the rs458741:C variant was strongly associated with extremely large VLDL, body composition, and the incidence of diabetes. This finding illuminates the strong genetic linkage between VLDL modification and insulin resistance.

### Gut microbiome imbalance

Vojinovic et al. (64) showed in a prospective population-based cohort of 2309 individuals that 32 microbial families and genera in gut microbiota were associated with size-defined subfractions of VLDL, HDL, serum lipid values, and glycolysis-related metabolites. Among the 32, 18 microbial families and genera were significantly associated with VLDL particles of various sizes (extra small, small, medium, large, very large, and extremely large) (64). Another recent study showed that, in healthy individuals, low microbiota diversity was associated with obesity, abdominal obesity, and low HDL-C level (65). These reports suggest that gut microbiota imbalance may be involved in the alteration of VLDL particle size. Thus, the source of altered VLDL particles is presumably the intestines, although the real origin of altered VLDL particles may be diet. In animals and humans, a high-carbohydrate diet results in the elevation of large TG-enriched VLDL particles, along with the enrichment of apoC proteins. Carbohydrate intake increases hepatic secretory rates of VLDL TGs without changing the secretion of apoB, which together lead to large and dense VLDL particles (66).

## Very-low-density lipoprotein particles in the non-fasting state carry a risk for atherosclerosis and atrial fibrillation

### Very-low-density lipoprotein particle changes in fasting and postprandial states

Postprandial hypertriglyceridemia is a hallmark of dyslipidemia in patients with type 2 diabetes. Recently, it has been suggested that postprandial dyslipidemia is equally as important as the estimation of lipids in the fasting state, particularly for patients with type 2 diabetes (67). Mora et al. (27) characterized lipoprotein particles according to size in fasting and non-fasting states by using NMR, noting similar results between LDL and HDL particles. However, compared with fasting VLDL, non-fasting large VLDL particles carried much higher risk for diabetes. In the Copenhagen General Population Study, in which NMR spectrometry was used to analyze the lipids of 9293 individuals, the results showed that VLDL and IDL particles contained one-third of plasma cholesterol in the non-fasting state (68). Postprandial TGs are carried by primarily chylomicron and VLDL remnants, which are ligands of the VLDL receptor involved in macrophage foam cell formation during the development of atherosclerosis (69).

### Correlation of postprandial very-low-density lipoprotein rather than fasting very-low-density lipoprotein with atrial cardiopathy

VLDL utilization serves as the major energy source for the heart. Under physiologic conditions, approximately 70% of the heart’s energy is derived from fatty acid oxidation (70). Lee et al. (71) showed that postprandial VLDL is independently correlated with atrial enlargement, indicating that postprandial VLDL is a risk factor for atrial fibrillation (Table 1). In a prospective study of individuals with MetS (*n* = 87) and without MetS (*n* = 80), they found that negatively-charged VLDL (2-h postprandial VLDL-χ, concentration in %), waist and hip circumferences, body mass index, and blood pressure were positively correlated with left atrial diameter. After adjusting for obesity and blood pressure, 2-h postprandial VLDL-χ, but not fasting VLDL, was independently correlated with left atrial diameter. Each 1% increase in VLDL-χ correlated with an incremental left atrial diameter increase of 0.23 cm. Nakajima et al. (72) showed that postprandial VLDL has a higher affinity to the VLDL receptor, with better internalization into cells than non-postprandial VLDL. With these findings in mind, postprandial modified VLDL has been suggested as a therapeutic target for atrial remodeling in patients with MetS (54).

VLDL composition, especially in the postprandial state, is influenced by meals and eating habits. Guerrero et al. (73) described the effects of a sucrose-enriched diet on elevated levels of VLDL-cholesterol and TGs, insulin resistance, and hepatic steatosis in male Wistar rats. In addition, Drorna et al. (74) reviewed the available evidence for the impact of high-fructose intake on health. In healthy individuals, the consumption of up to 1.5 g fructose/kg body weight per day for 4 weeks resulted in increased plasma TG concentrations (74). In addition to elevating TG levels, high fructose intake can induce hepatic steatosis, insulin resistance, and hyperuricemia (74). It is very likely that high fructose intake can alter VLDL particles with respect to size and TG richness. After a single high-fat meal, postprandial changes in TGs and VLDL can be significant in men with abdominal obesity compared with non-obese men (75). However, no such difference was observed between obese and non-obese women (75), suggesting sex-based differences in postprandial VLDL secretion during the reproductive stage.

## Therapeutic implications

### Nutritional intervention

In patients with existing cardiometabolic risks, 8-week nutritional intervention with a high polyphenol diet can significantly reduce the postprandial lipid content of large VLDL after a high-fat test meal (76). Another study showed that the consumption of a diet composed of fruit, avocado, whole grains, and trout for 8 weeks can reduce fasting insulin and VLDL and lower the postprandial increase in TGs and VLDL (77). With respect to the intake of fish, notable differences were seen in the NMR lipoprotein profile of the three main n-3 fatty acid subtypes: eicosapentaenoic acid (EPA), docosahexaenoic acid (DHA), and a-linolenic acid (ALA). Only a high intake of EPA significantly reduced VLDL particles and VLDL TGs (78). In addition, the reduction of apoCIII expression is believed to be the mechanism underlying the TG-lowering effects of omega-3 carboxylic acids, which contain 50–60% EPA and 15–25% DHA, as well as other active omega-3 free fatty acids (79). Fasting *per se* is beneficial for VLDL modification. In a study of 40 relatively healthy, middle-aged individuals, long-term fasting improved the postprandial lipid profile, especially with respect to the concentrations of large VLDL particles, which are significantly decreased after 7 and 14 days of fasting (80). Nevertheless, the impact of nutritional intervention on clinical cardiovascular outcomes warrants long-term observation and follow-up.

### Potential of other very-low-density lipoprotein-targeted therapies

In addition to nutritional intervention, synbiotic and probiotic supplements that improve gut microbiome imbalance have shown potential for decreasing serum VLDL-C levels (81). In addition, several oral anti-diabetic drugs have been identified that promote beneficial effects on VLDL metabolism. Pioglitazone, a PPAR-γ activator, was shown to facilitate LPL activity and promote the clearance of VLDL (82). Furthermore, glucagon-like peptide 1 (GLP-1) agonist reduced TG levels in the liver and the VLDL secretion rate (83).

Commonly used lipid-lowering drugs, although not specifically VLDL-targeted, have also been shown to help reduce VLDL. HMG-CoA reductase inhibitors (i.e., statins) reduce one-third of VLDL-TGs and more than 40% of apoCIII levels (84). In addition, peroxisome proliferator-activated receptor-α (PPAR-α) agonists (i.e., fibrates), which are prescribed primarily for managing hypertriglyceridemia, reduce VLDL-apoCIII levels, as well (84). Similar to selective estrogen receptor modulators, the first selective PPAR-α modulator (SPPARMα) LY-518674, which targets the receptor–cofactor binding profile of the PPARα ligand, modulates tissue- and gene-selective responses. In clinical phase II/III trials, this SPPARMα agonist reduced TG and apoCIII levels by about 50% (85). Proprotein convertase subtilisin-kexin type 9 (PCSK9) inhibitors, which reduce the degradation of LDL receptors and promote LDL uptake in the liver, also upregulate VLDL receptors and reduce VLDL levels. PCSK9 inhibitors have also been shown to preferentially modify the size and apolipoprotein composition of VLDL particles (86).

Several lipid-lowering agents are under development, including CETP inhibitor (87), microsomal triglyceride transfer protein (MTTP) inhibitor (88), and antisense oligonucleotides targeting the genes encoding apoB100 (88) and apoCIII (89). These therapeutics are currently being tested in clinical trials. Monoclonal antibody targeting ANGPTL3 has been shown to robustly reduce VLDL levels but at the expense of elevating LDL levels (90). In addition, ARO-ANG3 is an siRNA-based medication that inhibits the hepatic translation of *ANGPTL3* mRNA [102]. These new medications have the potential to produce favorable effects on VLDL structure and metabolism.

## Concluding remarks

**FIGURE 2:**
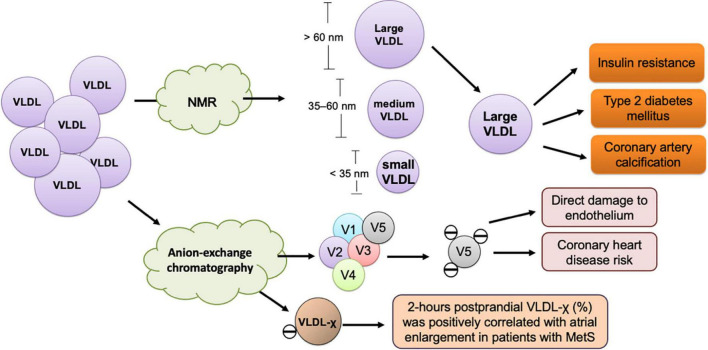
Size- and charge-defined subfractions of VLDL and their association with cardiometabolic diseases. The size-defined classification of VLDL according to particle diameter is performed using nuclear magnetic resonance (NMR) spectrometry. Large VLDL, which has a diameter larger than 60 nm, is associated with insulin resistance, type 2 diabetes mellitus, and coronary artery calcification. VLDL-χ or V5, the most negatively-charged subfraction of VLDL, is isolated and measured using anion-exchange chromatography. VLDL-χ or V5 causes direct damage to the endothelium and associated with coronary heart disease risk and atrial myopathy in metabolic syndrome (MetS).

Independent of LDL-C, VLDL’s atherogenic properties are associated with TG abundance, which largely affects particle size, apolipoprotein content alteration, electrical charge, and lipid composition, especially in the postprandial state (Figure 2). With adverse modification, VLDL facilitates ectopic lipid accumulation, which has been observed in the liver, heart, and skeletal muscles. To elucidate the pathogenic roles of VLDL in cardiovascular diseases, the issues of modification, in both fasting and postprandial states, should be taken into consideration. To improve adversely modified VLDL, nutritional intervention, especially through the reduction of fructose content in food, should be widely recommended, especially for patients with insulin resistance and cardiometabolic risks. However, interpreting data from only the size-based, charge-based, or apolipoprotein-based classified VLDL does not provide complete knowledge or information about lipids in health and diseases. To obtain a more comprehensive understanding of the lipid transport and metabolism process, methodologies are needed that can reflect the complex immunochemical and functional properties of all apolipoprotein-containing lipoproteins in the blood.

## Author contributions

H-CL contributed to the conceptualization of the study, participated in funding acquisition, and wrote the manuscript. AA and C-HC reviewed and edited the manuscript. All authors have approved the submitted version and agreed to be personally accountable for their own contributions.
